# TCM treatment of allergy induced by stainless steel implants for tibiofibular fracture: A case report 

**DOI:** 10.5414/ALX02095E

**Published:** 2019-12-30

**Authors:** Yansong Qi, Yi Ding, Baoge Liu, Yongsheng Xu

**Affiliations:** 1 Department of Orthopedics, Inner Mongolia People’s Hospital, Hohhot, Inner Mongolia,; 2 Department of Orthopedics, General Hospital of Chinese People’s Armed Police Forces (CAPF), and; 3 Department of Orthopedic Surgery, Beijing Tiantan Hospital, Capital Medical University, Beijing, China;; *YSQ and YD contributed equally to this work and share the first authorship

**Keywords:** external fixation, type IV allergy, TCM, metal allergy, contact dermatitis

## Abstract

Background: Metal allergy is frequently seen. Orthopedic metal implants, such as external fixators or other stainless implants, contain chromium, nickel, and molybdenum, which can cause type IV hypersensitivity. Case summary: A patient diagnosed with open comminuted tibiofibular fracture was treated with external fixation surgery, and she showed contact dermatitis and eczema-like symptoms 2 weeks postoperatively. She was then diagnosed as allergic to several metals by patch test and subsequently treated with traditional Chinese medicine (TCM), both orally and externally for 1 month. TCM treatment significantly alleviated the hypersensitive symptoms and made the patient bear the external fixator for 2 months until bone union. Conclusion: TCM therapy may be an effective treatment for external fixation-induced metal allergy and contact dermatitis.

**German version published in Allergologie, Vol. 42, No. 12/2019, pp. 559-566**

## Introduction 

Management open comminuted distal tibiofibular fractures is complex and always requires open reduction and fixation surgery [1]. The mid-to-lower segment of tibiofibular fracture is unstable, and outcomes are often bad. Complications can be delayed union and nonunion [2], infection, necrosis, and defect of skin and bone [3], especially when the fracture is open and comminuted. Thus, management of the distal tibiofibular fracture is complicated depending on different situations. External fixatation can provide rigid enough fixation and reduce infection, it can also promote bone union when the fracture is comminuted [4]; thus, open comminuted fracture is one of the relative indications of external fixation. 

External fixation systems contain many metal elements, such as percutaneous fixation pins, which are made of stainless steel and used for fixation of fracture segments. Besides iron, stainless steel also contains ~ 18% chromium, 15% nickel, and 3% molybdenum. To our knowledge, allergy to chromium, nickel, or cobalt is frequent in the general population [5, 6, 7]. However, research on metal allergy in the orthopedic field is limited, and orthopedists usually do not have much clinical experience in the management of metal implant allergy or hypersensitivity. 

Metal implant hypersensitivity is a delayed-type hypersensitivity (DTH, type IV allergy) involving activation of T-lymphocytes and releasing cytokine to attract macrophages [8, 9]. It often lead to various of skin lesions, including eczema, impaired wound and fracture healing, infection-mimicking reactions, and exudation [10]. In this paper, we presented a patient who was allergic to the metal of the external fixator, and we will share our experience on the clinical manifestation, diagnosis, and treatment strategy. 

Traditional Chinese medicine (TCM) has a wealth of experience in treating allergic diseases or hypersensitivity, and it has been used in China and other Asian countries, such as Korea and Japan, for hundreds of years [11]. The main component of TCM is herbal therapy, including oral and external administration. Several researches have indicated that TCM has the pharmacologic potential of treating hypersensitivity, such as allergic rhinitis [12] and atopic dermatitis [13]. In our case, the oral TCM medications included: Cortex Moutan (*Paeonia suffruticosa*, cortex part) and Red Moutan root (Paeonia suffruticosa, root part from the red plants), *Lonicera japonica* (honeysuckle), licorice (*Glycyrrhiza uralensis*) and honey-fried Herba Ephedrae (*Ephedra sinica *Stapf, honey-fried). Topical TCM medications included: phellodendri (cortex part) and Cortex Dictamni (*Dictamnus dasycarpus *Turcz, cortex part). All of them are recorded in the Pharmacopoeia of the People’s Republic of China (2010 Edition) and were reported to have immunoregulatory and anti-allergic effects through inhibiting T-lymphocyte activity or cytokines participating in DTH [14, 15]. 

## Case summary 

**Figure 1.**
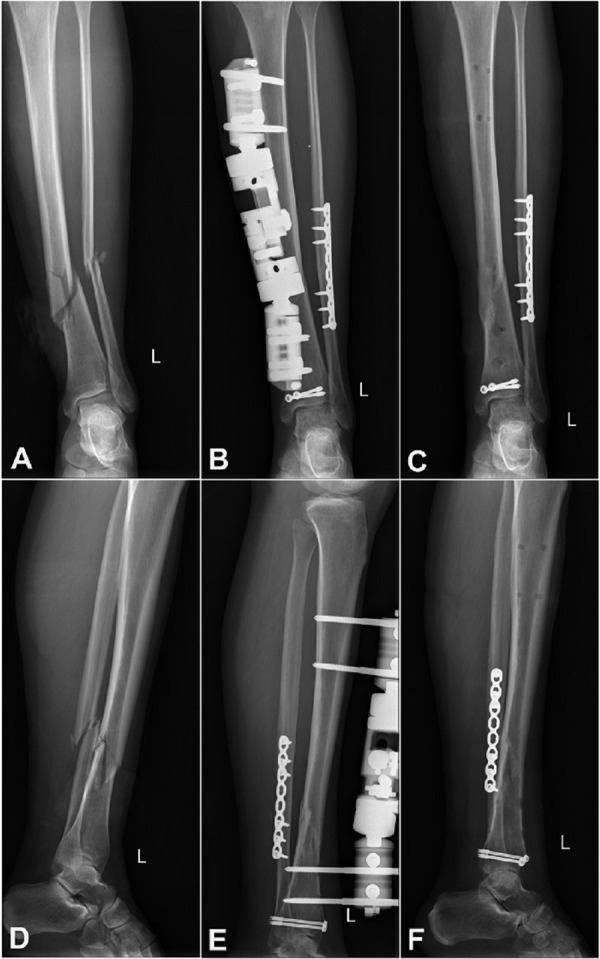
A, D: Comminuted fracture of tibia and fibula. B, E: X-ray showed some osteotylus across fracture line with good alignment 1 month after TCM treatment. C, F: Union of fracture without replacement after external fixation was removed.

A 40-year-old woman was diagnosed with open comminuted tibiofibular fracture and posterior malleolar fracture (Figure 1A, D) by the emergency department and transferred to our institution (Department of Orthopedics, Inner Mongolia People’s Hospital) 8 hours after injury. History: she was allergic to seafood, celery, sulfadiazine, and iodine, and denied chronic systemic diseases (she complemented her patient history with doubtable metal allergy when she came to hospital again after surgery). Considering that the distal tibiofibular fracture was open and comminuted, we performed a unilateral multifunctional external fixation (XY-034, Suzhou Yuxiang Medical Devices Co., Ltd., stainless steel) surgery for the tibia fracture and internal plate fixation for the closed fibula fracture (Figure 1B, E). 

**Figure 2.**
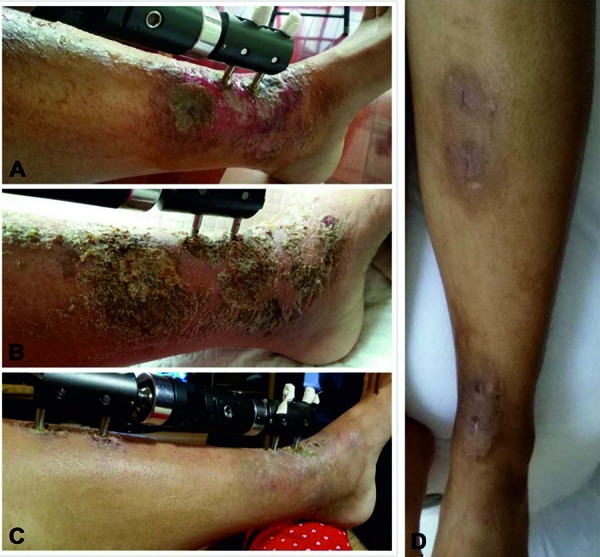
A: Skin allergic to external fixation screws showed eczema-like changes, small blisters, and exudate. B: 2 weeks after TCM therapy, the skin lesion showed scab formation (locally amplified to show scab formation and erythema relief). C: 4 weeks after TCM therapy, scab disappeared and the skin was restored to normal. D: The skin lesion was healed after removing external fixator.

The patient was hospitalized (General Hospital of Chinese People’s Armed Police Forces) again 2 weeks postoperatively due to skin lesions around the pin tracts of the external fixator. The skin lesions appeared as an eczema-like reaction and exudate with itch (Figure 2A). After further questioning, she complemented that she maybe also allergic to metal (“can’t wear necklace or watch for long hours in the summer”). Infection, psoriasis, and tinea were excluded, and then the patch test (for professional orthopedic use: BY-07-I and BY-07-II, Baiyi Yida Science and Technology Development Ltd., Beijing, China) was used to detect metal allergy [10]. 

**Figure 3.**
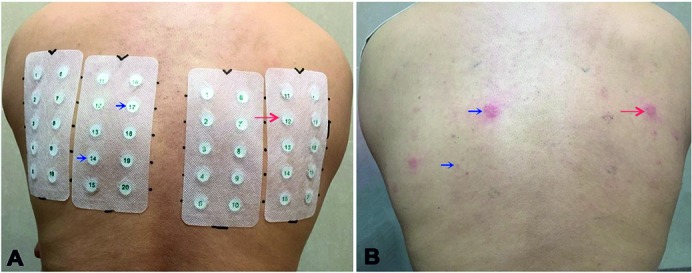
A: Patch test (BY-07-I and BY-07- II for orthopedic use, Baiyi Yida Science and Technology Development Ltd., Beijing, China): Blue arrows show No. 14 and 17 from left panel (BY-07-I), which correspond to potassium dichromate and nickel sulfate, respectively; red arrow shows No. 12 from right panel (BY-07-II), i.e., nickel nitrate. B: Arrows show a strongly positive allergic reaction (+++) to nickel and a doubtful reaction to chromium (±).

The patch test includes two panels, and each contains 20 spots for detecting metal allergens for professional orthopedics use. Each spot contains a metal salt or chemical allergen dissolved in white Vaseline. According to the International Contact Dermatitis Research Group (ICDRG) system [16], the patch test results were read after 48 and 72 hours. The results at 72 hours are presented in Table 1 and Figure 3. The test result showed a strongly positive reaction (+++) to nickel (No. 17 nickel sulfate from panel A (Figure 3B) (Table 1); No. 12 nickel nitrate from panel B, (Figure 3B) (Table 1) and a doubtable reaction (±) to molybdenum (No. 14 ammonium molybdate from panel B, (Figure 3B) (Table 1). 

Anti-allergic treatment was started with oral loratadine as well as compound vitamin and calcium gluconate intravenously for 2 weeks. However, the skin lesions persisted. Because routine treatment failed we chose to use TCM as anti-allergic therapy. TCM treatment included oral intake and topical use. The oral TCM prescription included: Cortex Moutan (*Paeonia suffruticosa*, cortex part) and Red Moutan root (*Paeonia suffruticosa*, root part from the red plants), *Lonicera japonica* (honeysuckle), licorice (*Glycyrrhiza uralensis*) and honey-fried Herba Ephedrae (*Ephedra sinica* Stapf, honey-fried). All of them were boiled out with pure water, and 100 mL of the decoction was administered twice a day. Topical TCM medications included: Phellodendri (cortex part) and Cortex Dictamni (*Dictamnus dasycarpus* Turcz, cortex part). Both of them were boiled out with pure water, and a gauze with decoction was wet packed on the skin for 20 minutes three times a day. 

TCM treatment was carried out for 4 weeks, and the patient did not receive any other treatment during this period. After the first week, her allergic skin symptoms and itch were alleviated. After the second week, the eczema-like skin lesion of contact dermatitis formed a scab (Figure 2B). The scab disappeared, and the skin recovered to the normal state at the fourth week (Figure 2C). The only side effect during the TCM treatment was mild diarrhea, which disappeared after treatment cessation. X-ray showed union of the fracture (Figure 1B, E) 1 month after TCM treatment, and the external fixation was removed (Figure 1C, F). The skin lesion was healed and the symptoms disappeared completely after the removal surgery (Figure 2D). 

## Discussion 

External fixation has traditionally been reserved for open fractures, polytrauma, and unstable fracture patterns. Unilateral external fixation is an effective treatment for open tibia fracture when bone is good and the defects are minor [17]. Generally, these external fixation systems contain chromium, molybdenum, and nickel, which can lead to metal allergy. Patch test is a detective test for allergens participating in type IV hypersensitive reaction. Chromium, molybdenum, and nickel are common metal allergens leading to type IV hypersensitivity (DTH). 

Although from the allergist’s point of view the best choice would have been to avoid the external fixator in our case, this open and comminuted distal tibiofibular fracture was complex and had to be treated thoughtfully. First, there is only little skin and soft tissue in the anteromedial aspect of the tibia bone, which can be subject to trauma or surgery [3]. Second, distal fractures at the mid-to-lower segment of the tibia are unstable and always accompanied by many complications, such as infection, delayed union, and nonunion [2], especially when the fracture is open or comminuted. Thus, from the orthopedist’s point of view, this tibiofibular fracture needed a rigid fixation. Considering the skin lesions was not appropriate for another open reduction and internal fixation surgery, and a calcaneus traction pin is also made of stainless steel, and a cast was not suitable due to the open wound and skin lesions. We decided to keep the external fixator because of its advantages for the open and comminuted fracture. Corticosteroids may cause nonunion or delayed union of fracture segments and skin, as well as infection in open comminuted fractures; thus, we decided not to use them neither externally nor systemically. What is more, there is no published evidence for using corticosteroids to treat metal allergy in open comminuted fractures [18]. 

TCM therapy significantly alleviated the hypersensitivity symptoms so that the patient could bear the external fixator for until bone union. Cortex Moutan and Red Moutan root can promote blood circulation and resolve hematoma [19, 20]. Paeonol is the mainly active ingredient in Cortex Moutan and the root, which is able to reduce TNF-α-induced cell migration and inflammation in DTH [15]. It has been proved that Paeonol has a significant anti-hypersensitive effect [21, 22, 23]. *Lonicera japonica* has anti-allergic effects, which has been used for treating skin inflammatory diseases including psoriasis and atopic dermatitis [24, 25, 26]. An in vitro study showed that *Lonicera japonica* can significantly downregulate TNF-α expression and inhibit proliferation, while it induced apoptosis in human keratinocyte cells [26]. This may explain the TCM effects on skin healing. *Lonicera japonica* also has anti-allergic effects. It was reported that *Lonicera japonica* alleviated IL-17/IL-23 secretion in monocytes and macrophages [26]. Licorice has immune-regulatory and anti-allergic activities through inhibiting T-lymphocyte activity [14]. Glycyrol is the natural anti-allergic ingredient extracted from licorice, which has been known as a new immunosuppressant as it can decrease DTH and inhibit IL-2 expression [27]. Besides, licorice was also used to reduce side effects of other herbs, which may be attributed to its detoxification effect of inducing nuclear factor erythroid 2-related factor 2 (Nrf2) expression and activating the Nrf2 signaling pathway [28]. Immunosuppressive effects of Herba Ephedrae were reported in many studies about TCM treatment for hypersensitivity, such as asthma [29], allergic rhinitis [30], and food-allergic diarrhea [31]. Herba Ephedrae works as an immunosuppressant, it can suppress T-cell over-activation and regulate immune homeostasis in hypersensitive disease through promoting Treg (regulatory T cells) development and proliferation [29]. 

Wet packing is a very effective method by which medicine can penetrate the skin. Golden Cypress (Cortex Phellodendri) has anti-allergy and anti-inflammatory effects on hypersensitive diseases such as cutaneous polyarteritis nodosa [32] and eczema [33]. An in vivo and in vitro study has found that Golden Cypress can suppress T-lymphocyte proliferation while enhancing Treg differentiation in some autoimmune diseases (DTH) [34]. Phellodendrine is the main biological immunesuppressor in Golden Cypress, and it suppresses the celluar immune response and the induction phase of DTH in animal studies [35]. Cortex Dictamni for external use can decrease inflammation of skin lesions and relieve itch [36]. There were two phenolic glycosides isolated from the water-soluble constituents of Cortex Dictamni, and both of them can notably inhibit T-cell proliferation in vitro [37]. What is more, Cortex Dictamni also has anti-inflammatory properties. Fraxinellone is the anti-inflammatory ingredient in Cortex Dictamni, it can negatively regulate IKK and ERK1/2 phosphorylation in macrophages and thus inhibit the NF-κB pathway and down-regulate iNOS and COX-2 expression [38]. Thus, external use of Golden Cypress and Cortex Dictamni may have anti-inflammatory and anti-hypersensitive functions for metal-induced contact dermatitis through these mechanisms. 

In summary, TCM had a significant effect on the contact dermatitis symptoms of type IV hypersensitive reactions induced by metal external fixation. Hence, the patient could bear the external fixator for long enough (2 months) until removal surgery. There were limitations in this paper: we did not perform a lymphocyte transformation test (LTT) or histologic testing according to the allergy diagnosis protocol [10], so there is no proof whether the TCM treatment can also eliminate or decrease the sensitization rather than only alleviate the symptoms. However, further pharmacology studies and clinical studies of TCM treatment for metal allergy are needed. 

## Conclusion 

Metal allergy is not rare among orthopedic patients. Patch tests should be performed when there is a history or even only doubtful history of metal allergy. External fixation metal-induced allergy is a type IV hypersensitivity (DTH), presenting as contact dermatitis. TCM treatment alleviated the hypersensitive symptoms of metal allergy, helping to bear the external fixator for enough time until bone union. TCM treatment may have anti-inflammatory and anti-hypersensitive effects on metal-induced DTH reactions. 

## Funding 

This study was supported by the District Science Fund Project of National Natural Science Foundation of China (No. 81560374). 

## Conflict of interest 

All authors declared that they have no competing interests. 

**Table 1.** Patch test and results at 72 hours.

Panel A		Panel B	
No. 1	1,4-Butanediol dimethacrylate (–)	No. 1	Aluminum nitrate (–)
No. 2	1,6-Hexanediol diacrylate (–)	No. 2	Ferrous chloride (–)
No. 3	2-hydroxyethyl acrylate (–)	No. 3	Ferric chloride (–)
No. 4	Ethyleneglycol dimethacrylate (–)	No. 4	Palladium chloride (–)
No. 5	N,N-Dimethyl-p-toluidine (+)	No. 5	Zinc sulfate (–)
No. 6	rosin (–)	No. 6	Manganese chloride (–)
No. 7	Methyl methacrylate (±)	No. 7	Silver nitrate (–)
No. 8	2,6-Di-tert-butyl-4-methylphenol (±)	No. 8	potassium dichromate (–)
No. 9	Clove oil (++)	No. 9	Cobalt chloride (–)
No. 10	paraformaldehyde (–)	No. 10	Copper sulfate (–)
No. 11	formaldehyde (–)	No. 11	Mercuric chloride (–)
No. 12	Benzocaine (–)	No. 12	Nickel nitrate (+++)
No. 13	thimerosal (–)	No. 13	Cadmium chloride (+)
No. 14	potassium dichromate (±)	No. 14	Ammonium molybdate (±)
No. 15	Palladium chloride (–)	No. 15	Titanium chloride (–)
No. 16	Copper sulfate (–)	No. 16	Gold chloride (–)
No. 17	Nickel sulfate (+++)	No. 17	Chloroplatinic acid ammonium (–)
No. 18	Silver nitrate (–)	No. 18	Indium trichloride (+)
No. 19	Titanium chloride (–)	No. 19	Tetrachloride iridium (–)
No. 20	Cobalt chloride (–)	No. 20	Control (–)

The results were read 72 hours after patch test according to the ICDRG scoring system [16]. The vehicle was white Vaseline, and 25 μL of detecting paste was applied to each spot. ICDRG scoring with six levels: negative (–), doubtful (±), mild erythema (+), moderate erythema with small blister (++), severe erythema or swollen with great blister (+++), severe erythema, and irritation reaction (IR).
